# Ingredients for Success: What Clients and Informal Caregivers Value in Depression Treatment A Concept Mapping Study

**DOI:** 10.1002/jclp.70092

**Published:** 2026-01-20

**Authors:** Reine M. D. Ramaekers, Dyllis A. van Dijk, Marja Y. Veenstra, Koen R. J. Schruers, Suzanne C. van Bronswijk, Nicole K. Leibold

**Affiliations:** ^1^ Research, Institute for Mental Health and Neuroscience, Faculty of Health, Medicine and Life Science Maastricht University Maastricht the Netherlands; ^2^ Parnassia Psychiatric Institute, PsyQ The Hague the Netherlands; ^3^ Burgerkracht Limburg Sittard the Netherlands; ^4^ Topclinical Center for Anxiety, OCD and Trauma, Mondriaan Mental Health Center Maastricht/Heerlen the Netherlands; ^5^ Maastricht University Medical Center+ Maastricht the Netherlands

**Keywords:** clients, concept mapping, depression treatment, informal caregivers, mixed methods, treatment success

## Abstract

**Objectives:**

Effective treatments are available for major depressive disorder; however, treatment efficacy is less pronounced in real‐life settings compared to research. One reason for this discrepancy may be that treatment outcomes assessed in research do not fully reflect domains important to treatment recipients: clients and their informal caregivers. Moreover, studies often struggle to assess the impact of process‐related factors on treatment success. More research incorporating client and informal caregiver perspectives is therefore needed to identify what they consider essential for a successful treatment.

**Methods:**

Group Concept Mapping was employed to elicit insights from clients and informal caregivers regarding their experiences with depression treatment. Twenty‐one participants brainstormed in response to the statement: “Successful depression treatment requires…”. Subsequently, 32 participants sorted the identified factors into meaningful groups and rated their importance to treatment success.

**Results:**

Participants generated 79 unique responses in the brainstorm. They sorted these responses into 10 clusters: “The client”, “Treatment process”, “Treatment organisation”, “Interaction client clinician”, “The clinician”, “Clinician′s adherence to good practice”, “Drug treatment”, “Pre‐condition”, “Supporting activities”, and “Supportive work and home life”. Most clusters were considered important.

**Conclusion:**

These findings support the idea of using multivariate and multimodal models for understanding treatment success. Participants attributed the therapeutic alliance as more clinician‐driven than client‐driven. A combination of factors related to treatment organization, treatment elements, and guideline adherence emerged as a core concept to successful treatment. Recommendations include increasing clinician awareness of perceptions of the therapeutic alliance and utilizing the findings to guide treatment discussions.

## 1 Introduction

Various effective treatments are available for major depressive disorder (MDD). People receiving psychological interventions experience a greater reduction in depressive symptoms than untreated controls (Cuijpers et al. 2023). In the short term, those receiving antidepressive medication experience about the same effectiveness as those receiving psychological treatment (Cuijpers et al. 2020). The combination of psychotherapy and pharmacotherapy seems to be the most effective treatment (Cuijpers et al. 2020). The effectiveness of initial treatment is important because inadequate treatments contribute to persistent depressive symptoms, and chronic depression makes future treatment response less likely (Rush et al. 2006). Therefore, understanding what makes treatment effective is important for improving treatment response. To better understand the factors contributing to depression treatment outcomes, research has increasingly focused on identifying baseline characteristics that may predict response or non‐response to therapy across different populations. In a comprehensive review, Kessler et al. (2017) identified 30 baseline factors associated with treatment outcome, including age, sleeping quality, and childhood maltreatment. Additionally, baseline severity, co‐morbid anxiety, and executive dysfunction were predictors for worse treatment outcomes for clients over the age of 60 (Tunvirachaisakul et al. 2018). Similarly, Perlman et al. (2019) showed that older age increases the risk of non‐response.

With the limited *efficacy* of depression treatments demonstrated in research settings, with only about half of individuals recovering after treatment (National Health Service 2018), strong evidence indicates that their *effectiveness* is even less pronounced in real‐world clinical practice (Van Der Lem et al. 2012a). This, and other factors, could contribute to the ‘treatment‐prevalence paradox′ (Ormel et al. 2022), which is the notion that even though the quality of depression treatment has increased, and treatments are more widely accessible, the prevalence of depression has not decreased. The gap between efficacy observed in research settings and effectiveness in clinical practice might be due to the fact that clients in real‐life might not resemble clients included in RCTs (Stirman et al. 2003; Van der Lem et al. 2012b) and that the protocols implemented in clinical practice are not as specified and monitored as treatment in RCTs, exemplified by Interpersonal Psychotherapy rarely having a frequency of twice a week in practice though this is shown to be more effective in RCTs (Bruijniks et al. 2020; Ormel et al. 2022).

Another concern regarding the clinical translation of treatment studies into real‐world settings is whether the outcome domains currently assessed to determine effectiveness truly matter to those directly affected by depression—namely, the clients and their informal caregivers. Zimmerman et al. (2022) put forward that focusing mainly on symptom reduction is inadequate for understanding true treatment success. In addition, clients and informal caregivers consider broader outcomes in addition to symptom severity to be important for treatment success, including social and professional functioning and being able to manage the depression (Chevance et al. 2020; Kan et al. 2020; Rost et al. 2023a; Veal et al. 2024). This suggests that besides RCTs not generalizing well, they also might not reflect real‐life well because the measures of treatment success that are employed do not capture the full picture of treatment success through the eyes of clients and informal caregivers.

Beyond the outcome domains used to assess treatment success in studies, certain factors or variables have been identified as contributing factors, though research commonly focuses on (static) predictors measures at the start of treatment (e.g., age, comorbidities, marital status, and socioeconomic status; Kessler et al. 2017; Perlman et al. 2019; Tunvirachaisakul et al. 2018). However, some researchers suggest that dynamic factors that develop during treatment, the so‐called mechanisms of change, are key to treatment success (Lemmens et al. 2016). An example of such a mechanism is the quality of the therapeutic alliance, which is positively correlated to treatment success for several therapies (Arnow et al. 2013; Barber et al. 2014; Leibovich et al. 2020; Zilcha‐Mano et al. 2015). However, it is difficult to measure mechanisms of change during RCTs (Cuijpers et al. 2019; Lemmens et al. 2016).

To improve the effectiveness of treatments, it is, therefore, essential to include input from clients and informal caregivers in the research process and study how they perceive the ongoing processes of treatment and its success. However, the perspective of clients and informal caregivers in depression treatment outcome research has received less attention and should receive more attention in research (Cuijpers 2019). Studies that do include this perspective feature the importance of the therapeutic relationship in which the client feels, among others, supported and validated (Ladmanová et al. 2022; Levitt et al. 2016; Marren et al. 2022). Next to the therapeutic relationship, clients mention the importance of working with clinicians on clarifying and resolving specific problems, us specific techniques, and providing space to experience difficult emotions (Constantino et al. 2022; De Smet et al. 2020). Specifically in the treatment of depression, van Grieken et al. (2016) found that clients, carers, and professionals list factors related to a professional therapist, treatment content, a structured treatment process, and treatment organization as helpful in depression treatment.

Despite these promising results we still know little about what clients and, even more, informal caregivers define as factors contributing to treatment success. The current study therefore aims to collect and describe factors that clients and informal caregivers regard as necessary ingredients for successful depression treatment. By combining qualitative and quantitative methods, experiences of clients and informal caregivers can be systematically collected in a way that includes experiences that may be difficult to capture by numbers alone. The current study can therefore serve as a bridge between the RCTs and real‐life. Such understanding would not only shine light on the treatment‐prevalence paradox but could also contribute to a more effective approach to treatment.

## 2 Materials & Methods

### 2.1 Participants

Participants received depression treatment or cared for someone receiving depression treatment in the past 3 years. Exclusion criteria were: 1) not having sufficient proficiency in either the English or Dutch language, 2) being under the age of 18, and 3) having received treatment for bipolar depression, as these treatments typically differ from those for unipolar depression (Grande et al. 2016).

Participants were recruited by various means. Flyers and posters were distributed physically at Maastricht University, in waiting rooms of Dutch mental health care institutions, and general physicians′ offices. Online, information about the study was distributed through social media. Additionally, information about the study was shared with local Dutch depression peer support groups via the Dutch Depression Society (the “Depressie Vereniging”).

Prior to the start of the study, ethical approval was obtained from the Ethics Review Committee Psychology and Neuroscience at Maastricht University. Before participation, participants gave written or online informed consent. Participants were rewarded for their participation with a financial voucher of €7.50 per hour and a minimum of €5.00.

### 2.2 Group Concept Mapping

Group concept mapping (GCM) is a method particularly suited for gathering input from stakeholders on their experiences (Kane and Trochim 2007). GCM includes participants and stakeholders in the design of the study, the gathering of the data, and the interpretation of the results (Kane and Trochim 2007). Additionally, GCM also involves asking participants to structure the factors they come up with by sorting and rating them. GCM consists of multiple steps: 1) preparation, 2) idea synthesis, 3) sorting and rating, and 4) analysis (Kane and Trochim 2007; Trochim 1989). The steps are described in detail below.

#### 2.2.1 Preparation

As the first step in the concept mapping process, a small expert group was invited to help prepare for the study. The expert group existed of two of the authors of the present paper (RR, researcher, and SvB, researcher and psychiatrist), a psychiatrist with expertise in mood disorders, an expert of a local consultancy in citizen participation named “Burgerkracht Limburg”, a member of a local depression peer support group associated with the Depression Society, and an unaffiliated informal caregiver. The expert group formulated a focus prompt that served as the basis for this concept mapping study. The focus prompt is meant to help participants brainstorm and generate ideas about the study′s topic. The focus prompt for this study was: “Successful depression treatment requires …”. The focus prompt was translated to Dutch.

#### 2.2.2 Idea Synthesis

As the second step in the process, participants were asked to brainstorm as many statements as possible, completing the focus prompt. Data for the brainstorming activity was gathered in two ways: an in‐person session and via an online web server called GroupWisdom (groupwisdom 2021).

An in‐person session was arranged with a local depression peer support group associated with the Depression Society. Participants in this session were introduced to the study in a short, in‐person introductory meeting. In a following meeting dedicated to the brainstorming activity, participants got a more elaborate explanation of the procedure. Participants filled out a short demographic questionnaire and then engaged in a 60‐minute brainstorming session led by RR. All participants were instructed that a successful depression treatment could include the reduction of symptoms, the improvement of quality of life, and the increase of psychological well‐being. The session was audio‐recorded and was completed in Dutch.

For online participation, participants were screened via a telephone interview to verify eligibility. Participants then got access to the web server GroupWisdom (groupwisdom 2021), where they filled out their demographic details and engaged in an asynchronous brainstorm on the focus prompt. Participants could see answers that other participants had given to the focus prompt and could add their own answers to this list. All participants were instructed that a successful depression treatment could include the reduction of symptoms, the improvement of quality of life, and the increase of psychological well‐being. They could choose to brainstorm in English and Dutch.

After closing the brainstorming activity, the list of all answers to the focus prompt was analysed by RR and SvB. In line with GCM methodology, any duplicates were taken out. According to a code wording system, unique statements were identified and synthesized into a final list (Kane and Trochim 2007).

#### 2.2.3 Sorting and Rating

In the third step, participants were asked to sort and rate the final statement list that resulted from the idea synthesis. Participants partially overlapped with the brainstorm, but new participants were also welcomed. Participants were first instructed to group statements into piles in a way that made sense to them, sorting statements together that were related in meaning. They were instructed not to include a “miscellaneous pile”, and not to pile statements according to importance. Participants were then asked to rate the statements in answer to the question: “How important is this statement toward improving depression treatment outcomes?” on a four‐point Likert scale, with 0 being “relatively unimportant”, 1 being “a little important”, 2 being “moderately important”, and 3 being “very important”.

As in the idea synthesis step, an in‐person session took place alongside online data collection. In‐person, the same local depression peer support group participated in a 90‐minute session. Online, people could participate again via GroupWisdom (groupwisdom 2021). Online sorting and rating also took about 90 min.

#### 2.2.4 Analyses

In the fourth step, Concept Systems software (groupwisdom 2021) was used to analyse the data from the sorting and rating procedure. First, multidimensional scaling was applied to create a point map with all statements plotted as points on the map. On this point map, distance represents a degree of similarity or conceptual overlap as seen by the participants, where small distances indicate that participants sorted the statements more often together in a pile than long distances and vice versa (Kane and Trochim 2007). A similarity matrix cut‐off score of 1 was applied, meaning that if two statements were only grouped together by one participant, this grouping was not taken into account in creating the point map. Second, hierarchical cluster analysis was applied to form clusters from the point map. Third, the choice for the final cluster solution and the titles of the clusters used in subsequent analyses were discussed within the expert group. Fourth, ladder graphs and go‐zones were developed from the data to compare importance ratings between participants with different symptom severity and different treatment experiences. Ladder graphs show different cluster importance ratings between different participant groups (Kane and Trochim 2007). Go zones show how individual statements might have been rated differently by different types of participants (Kane and Trochim 2007).

## 3 Results

### 3.1 Participants

Demographic details from participants are summarized in Table 1. In total, 21 participants with a mean age of 35.57 years (*SD* = 10.80) contributed to the brainstorming activity. Thirty‐two participants started the sorting activity (*M*_age_ = 38.03, *SD* = 12.66), four participants did not finish it, and sorting data from two participants were rejected because they did not follow the rules for sorting, which are explained in the methods section.

**Table 1:** Demographic details of participants brainstorming, and sorting and rating.

	Brainstorm (*N* = 21)	Sorting and rating (*N* = 32)
Age in years, *M (SD)*	35.57 (10.80)	38.03 (12.66)
Gender, *n* (%)		
Woman	12 (57.14)	20 (62.50)
Man	7 (33.33)	9 (28.13)
Missing	2 (9.52)	3 (9.38)
Educational level, *n* (%)		
High school degree	5 (23.81)	4 (12.50)
Vocational degree	3 (14.29)	3 (9.38)
Associate degree	3 (14.29)	4 (12.50)
University degree	10 (47.62)	20 (62.50)
Other	0 (0)	1 (3.13)
Experienced severity of symptoms, *n* (%)		
No	6 (28.57)	12 (37.50)
Mild	6 (28.57)	9 (28.13)
Moderate	7 (33.33)	11 (34.38)
Severe	2 (9.52)	0 (0)
Treatment experience in the past 3 years, *n* (%)		
None	3 (14.29)	7 (21.88)
Psychotherapy only	2 (9.52)	3 (9.38)
Antidepressive medication only	1 (4.76)	2 (6.25)
Combination of psychotherapy and antidepressive medication	15 (71.43)	20 (62.50)

### 3.2 Statements

During the brainstorming phase, 254 statements were collected. After analysing these statements, a final statement list consisting of 79 unique statements was synthesized. A list of these statements can be found in Table 2.

**Table 2:** Clusters, statements, and importance ratings.

Cluster solution	Statements		Cluster/Statement rating, *M* (*SD*)	
1 The client			**3.36**	
	1	the client accepting the diagnosis	3.55 (0.62)	
	2	activation of the client	3.41 (0.67)	
	5	capacity from the client to confront their own weaknesses	3.17 (0.75)	
	6	commitment from the client	3.66 (0.66)	
	7	Resilience from the client	2.97 (0.85)	
	8	a motivated client	3.54 (0.68)	
	9	That the client has insight that they are ill	3.31 (0.79)	
	14	a client that adheres to agreements made during treatment	3.48 (0.72)	
	53	hope for improvement from the client	3.21 (0.85)	
	66	progress	3.34 (0.71)	
**Cluster solution**	**Statements**		**Cluster/Statement rating**, *M* (*SD*)	
2 Treatment process			**3.15**	
	13	giving the client a clear role in treatment	3.28 (0.78)	
	35	frequent contact during all steps of treatment	3.34 (0.71)	
	50	clear goals for the client	3.24 (0.67)	
	51	evaluating goals	3.31 (0.65)	
	59	a choice of treatments when the current treatment does not work for or fit with the client	3.38 (0.72)	
	62	trying out different therapies	2.76 (0.86)	
	67	keeping to treatment guidelines and protocol	2.55 (0.97)	
	72	the client making their own treatment choices	3.21 (1.00)	
	77	no predetermined end date of the treatment	2.90 (1.03)	
	78	continuity between treatments	3.52 (0.68)	
**Cluster solution**	**Statements**		**Cluster/Statement rating**, *M* (*SD*)	
3 Treatment organisation			**3.30**	
	31	a multidisciplinary team	2.90 (0.84)	
	32	that the therapist is part of a clinical network to rely on their expertise and treatment options	2.90 (0.96)	
	34	good communication about treatment steps	3.52 (0.56)	
	38	good diagnostics	3.55 (0.77)	
	55	the possibility to change clinician if there is no appropriate match	3.66 (0.54)	
	58	monitoring (checking up how the client is doing)	3.31 (0.79)	
**Cluster solution**	**Statements**		**Cluster/Statement rating**, *M* (*SD*)	
4 The clinician			**3.17**	
	16	a therapist that tells the client when they do not know something	3.17 (0.79)	
	18	an empathic therapist	3.41 (0.72)	
	19	an open attitude from the clinician	3.62 (0.67)	
	20	vulnerability from the clinician	2.36 (0.93)	
	21	knowledge through their own experience from clinician	2.24 (1.13)	
	22	self‐reflection from the clinician	3.14 (0.86)	
	24	a therapist who has clinical experience with treating clients	3.21 (0.89)	
	25	flexibility from the therapist	3.24 (0.77)	
	26	a therapist with patience	3.59 (0.77)	
	27	a clinician who is not focused on the computer during treatment	3.24 (0.93)	
	28	cultural understanding from the therapist towards the client	3.24 (0.90)	
	23	well‐educated clinician(s)	3.55 (0.72)	
**Cluster solution**	**Statements**			**Cluster/Statement rating**, *M* (*SD*)
5 Interaction client clinician				**3.42**
	15	trust between client and clinician		3.86 (0.43)
	17	mutual respect between clinician and client		3.90 (0.30)
	29	good communication between employees of the clinician′s office		2.52 (1.04)
	56	timely evaluation of the match between client and clinician		3.28 (0.69)
	57	a good match between client and clinician		3.69 (0.65)
	76	addressing possible redirection of feelings in the communication between clinician and client (transference) in a safe way during treatment		3.28 (0.69)
**Cluster solution**	**Statements**			**Cluster/Statement rating**, *M* (*SD*)
6 Clinician′s adherence to good practice				**3.29**
	30	contact between the clinician and client′s other health care providers		2.93 (0.83)
	37	discussing the diagnosis with the client		3.59 (0.49)
	47	evaluating the cause of the depression		3.38 (0.81)
	61	informing about different therapy options		3.21 (0.85)
	63	attention for the client′s physical health		3.31 (0.83)
	65	paying attention during treatment to things that are nice instead of just symptoms		2.83 (0.83)
	69	adequate psychoeducation for the client		2.96 (0.91)
	71	a relapse prevention plan		3.38 (0.85)
	75	discussing practical tools on how to cope with depression		3.69 (0.53)
	79	a short waiting time		3.41 (0.89)
	36	treatment for other diagnoses in combination with depression treatment		3.14 (0.86)
	48	evaluating the triggers of the depression		3.66 (0.48)
**Cluster solution**	**Statements**			**Cluster/Statement rating**, *M* (*SD*)
7 Drug treatment				**3.54**
	33	communication about expectations of medication with the client		3.55 (0.62)
	46	regular critical evaluation of the need to take or not take certain medication		3.55 (0.56)
	73	a safe space to talk about side effects		3.52 (0.77)
**Cluster solution**	**Statements**			**Cluster/Statement rating**, *M* (*SD*)
8 Pre‐condition				**1.84**
	39	low severity of depression prior to treatment (i.e. having a mild depression before treatment)		1.79 (1.03)
	40	high severity of depression prior to treatment (i.e. having a severe depression before treatment)		1.90 (1.09)
**Cluster solution**	**Statements**			**Cluster/Statement rating**, *M* (*SD*)
9 Supporting activities				**2.82**
	3	activities that help the client reconnect to their bodies		2.86 (0.74)
	10	opportunity for the client to focus on themselves		3.36 (0.89)
	49	exercise (a client that physically exercises)		3.10 (0.88)
	60	reconnection of the client to nature		2.24 (1.01)
	64	discussing activities and things that the client can take energy from in their daily life		3.31 (0.70)
	70	a client who reads about depression		2.07 (0.87)
**Cluster solution**	**Statements**			**Cluster/Statement rating**, *M* (*SD*)
10 Supportive work and home life				**2.96**
	4	helping the client find back interests such as gardening, cooking together, pottery or other creative activities		3.14 (0.87)
	11	that the client has (prospect of) work, paid or voluntarily		2.59 (0.89)
	12	that the client has (prospect of) financial stability		3.00 (0.91)
	41	time‐off from employer to receive treatment		2.97 (1.00)
	42	having an understanding employer		3.03 (0.93)
	43	decreasing stress in the client′s home environment		3.38 (0.72)
	44	a safe home environment		3.45 (0.72)
	45	as little stigmatization as possible from the client′s environment		3.28 (0.78)
	52	professional support in the home environment		2.59 (1.07)
	54	involving loved ones		2.45 (0.97)
	68	involving close family members or friends in psychoeducation		2.38 (0.93)
	74	a stable social safety network around the client		3.24 (0.82)

### 3.3 Cluster Map

**Figure 1:**
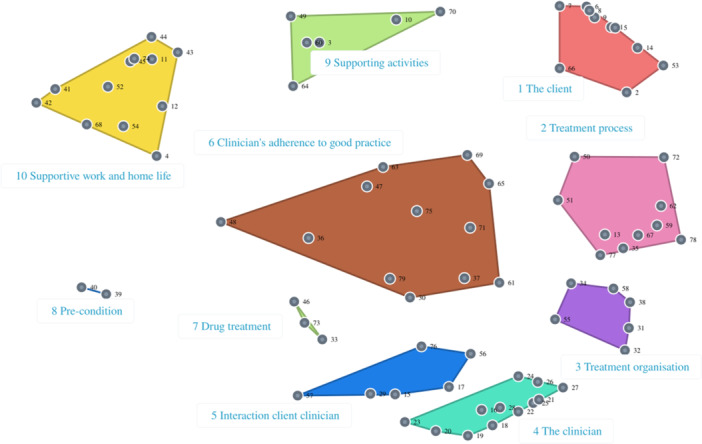
Cluster map of factors necessary for successful depression treatment.

Figure 1 shows the cluster map that resulted from the hierarchical cluster analysis and the consultation of the expert group. The point map had a stress value of 0.27. This is a goodness‐of‐fit measure, which is in line with stress values observed in other GCM studies and indicates a good internal validity of the data (Rosas and Kane 2012). The clusters were given titles based on participants′ input. The cluster titles were: “The client”, “Treatment process”, “Treatment organisation”, “Interaction client clinician”, “The clinician”, “Clinician′s adherence to good practice”, “Drug treatment”, “Pre‐condition”, “Supporting activities”, and “Supportive work and home life”. Three statements were moved from their original cluster to a cluster with a better fit in consultation with the expert group. Statement 17 (“mutual respect between clinician and client”) was moved from “The clinician” to “Interaction client clinician”. Statement 48 (“evaluating the triggers of the depression”) and statement 36 (“treatment for other diagnoses in combination with depression treatment”) were moved from “Drug treatment” to “Clinician′s adherence to good practice”. Table 2 details which statements are located in each cluster.

### 3.4 Cluster Locations

The location of each cluster can also give an indication as to how concepts are related to each other. The cluster map in Figure 1 shows “Clinician′s adherence to good practice” as a centre cluster. A cluster in the centre typically indicates that this concept is either at the core of the factors identified by participants or is a cluster of factors that did not clearly fit with any of the other clusters (Kane and Trochim 2007). This was discussed with the expert group, who identified this cluster to be at the core of the other factors. Another interesting observation is that “Interaction client clinician” is located closer to the cluster related to the clinician than the client.

### 3.5 Importance Ratings

#### 3.5.1 Clusters

The cluster “Drug treatment” was rated as most important, with an average importance rating of 3.54. The cluster “Pre‐condition” was rated least important with an average importance rating of 1.84. Overall, clusters had a high average importance rating, with seven out of ten clusters having an importance average rating higher than three. Figure 1 shows all clusters and Table 2 details the average importance ratings per cluster.

### 3.6 Statements

The importance ratings per statement were skewed to the left, meaning that many factors were rated as very important. The standard deviations of the importance ratings were normally distributed. The three statements with the highest average importance rating were all clustered in “Interaction client clinician”. These statements were: “mutual respect between clinician and client” (*M* = 3.90, *SD* = 0.30), “trust between client and clinician” (*M* = 3.86, *SD* = 0.43), and “a good match between client and clinician” (*M* = 3.69, *SD* = 0.65). The ten most important statements are shown in Table 3.

**Table 3:** Ten statements with highest average importance rating.

Statement number	Statement	Cluster name	Average importance rating, *M* (*SD*)
17	Mutual respect between clinician and client	Interaction client clinician	3.90 (0.30)
15	Trust between client and clinician	Interaction client clinician	3.86 (0.43)
57	A good match between client and clinician	Interaction client clinician	3.69 (0.65)
75	Discussing practical tools on how to cope with depression	Clinician′s adherence to good practice	3.69 (0.53)
6	Commitment from the client	The client	3.66 (0.66)
48	Evaluating the triggers of the depression	Clinician′s adherence to good practice	3.66 (0.48)
55	The possibility to change clinician if there is no appropriate match	Treatment organisation	3.66 (0.54)
19	An open attitude from the clinician	The clinician	3.62 (0.67)
26	A therapist with patience	The clinician	3.59 (0.77)
37	Discussing the diagnosis with the client	Clinician′s adherence to good practice	3.59 (0.49)

### 3.7 Ladder Graphs and Go‐zones

Ladder graphs and go‐zones were created to determine whether there were any differences in importance ratings between different groups of participants. Ladder graphs show differences in cluster ratings. Go‐zones show differences in statement ratings.

#### 3.7.1 Symptoms

**Figure 2:**
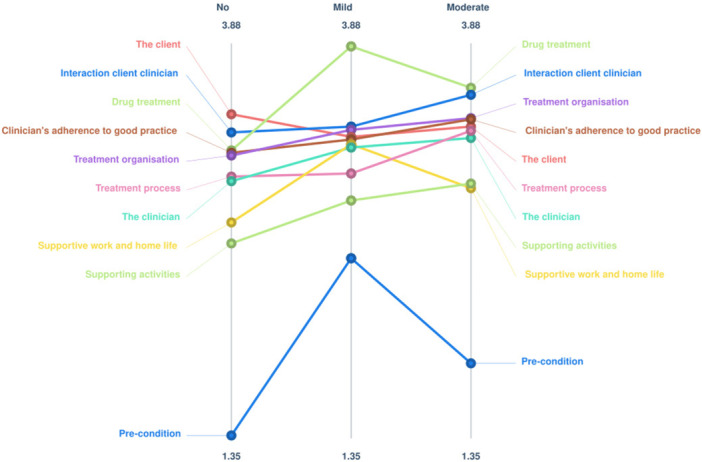
Ladder graph comparing average importance ratings for different symptom severities.

Figure 2 shows the ladder graph comparing average importance ratings of clusters from participants with no, mild, or moderate symptoms. Generally, ratings were very similar, with a strong correlation of *r* = 0.80 between the no symptoms group (*n* = 10) and the mild symptoms group (*n* = 8), a strong correlation of *r* = 0.84 between the mild symptoms group and the moderate symptoms group (*n* = 11), and a strong correlation of *r* = 0.97 between the moderate symptoms group and no symptoms group. The go‐zone confirmed that statements were rated in the same trend by people experiencing and not experiencing symptoms. The strong diagonal line indicates statements were rated as similarly important by the two groups. This go‐zone can be found in Appendix A.

#### 3.7.2 Treatment Experience

**Figure 3:**
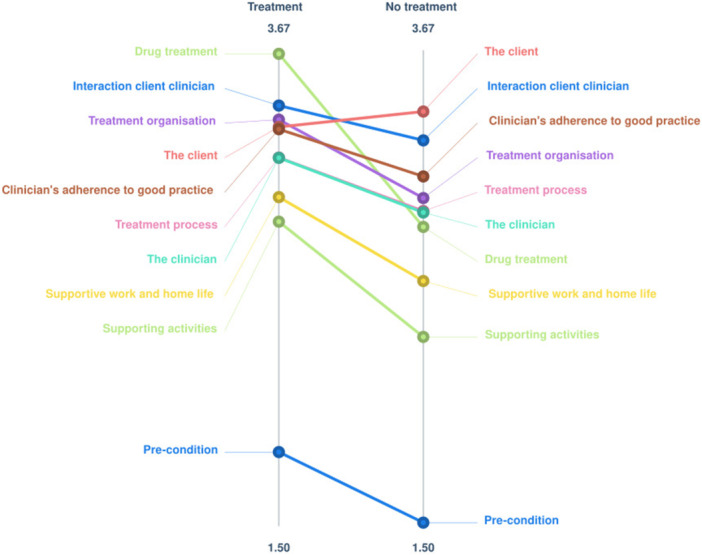
Ladder graph comparing average importance ratings by having had treatment.

Figure 3 shows the ladder graph comparing average importance ratings of clusters from participants who had received treatment (*n* = 23) in the past 3 years to participants who had not received treatment (*n* = 5), i.e. clients versus informal caregivers respectively. Generally, ratings are very similar. There is a strong correlation of *r* = 0.91 between the average ratings. There seems to be a trend that clients rated drug treatment as a more important factor for successful treatment than informal caregivers. Due to the small sample size, no ad‐hoc statistical tests were performed. The go‐zone in Appendix B confirms that most statements are considered similarly important by the two groups, visible by the strong diagonal line.

**Figure 4:**
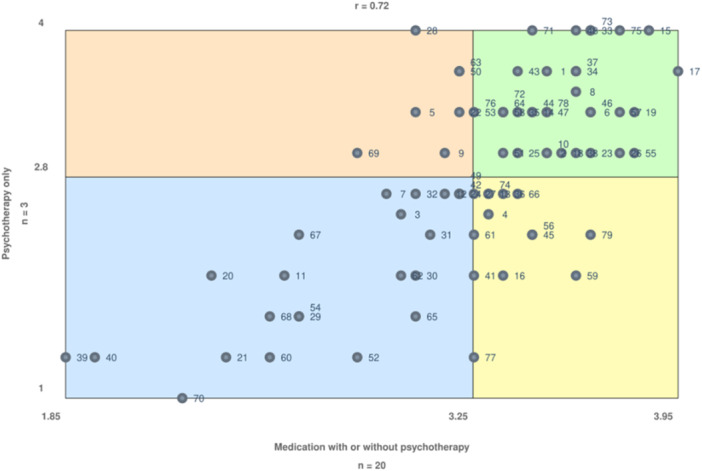
Go‐zone comparing average statement importance for different treatment experiences.

To zoom in particularly on whether treatment modality influences the importance of specific factors, we compared statement importance ratings from participants who received treatment with or without medication. To visualize this, we composed a go‐zone graph, comparing statement ratings for participants who received psychotherapy only and participants who received antidepressant medication with or without psychotherapy. This go‐zone is shown in Figure 4. The diagonal line that the dots follow indicates that, generally, statements are rated very similarly. Three statements do not follow this diagonal. Participants who received antidepressive medication seem to have rated statement 79 (“a short waiting time”), statement 59 (“a choice of treatments when the current treatment does not work for or fit with the client”), and statement 77 (“no predetermined end date of the treatment”) as more important than participants who received treatment but without antidepressive medication.

## 4 Discussion

The current study combined a qualitative and quantitative approach to comprehensively assess which key factors clients and informal caregivers perceive as relevant to treatment success in depression, which can shine light on the treatment‐prevalence paradox, but could also contribute to a more effective treatment approach. Clients and informal caregivers identified 79 factors that they consider important to successful depression treatment, rated these factors, sorted them into 10 clusters. Interesting findings were that the “Interaction client clinician” was more closely positioned to the clinician‐ than to the client‐cluster and the cluster “Clinician′s adherence to good practice” seemed to be at the core of important factors. Additionally, on average, clusters were rated as relatively important. Specifically, the three most important factors were: mutual respect, trust, and a good match between client and clinician. Moreover, when focusing on people who had received treatment compared to people without (clients vs. informal caregivers), it appeared that people with treatment judged “Drug treatment” as more important. Additionally, people who received treatment with antidepressant medication seemed to particularly judge the factors “a short waiting time”, “a choice of treatments when the current treatment does not work for or fit with the client”, and “no predetermined end date of the treatment” as more important than people who had only received psychotherapy.

Overall, clients and informal caregivers indicated many of the identified factors to be relatively important to successful treatment, which becomes apparent in the skewed distribution towards high values of statement importance ratings. These results corroborate the notion and provide several variables to include in a multivariable model to better explain treatment outcomes (Cohen and DeRubeis 2018; Furukawa et al. 2020; Furukawa et al. 2019; Perlis 2013). An advantage of a multivariable model is that it might better illustrate the complexity and multifactorial nature of MDD and its treatment as it is in the real‐world, which is difficult to capture with stringently organized RCTs (Ormel et al. 2022). While some noteworthy attempts have been made in this respect, multivariable models should still be approached with caution (Rost et al. 2023a; Sajjadian et al. 2021; Van Bronswijk et al. 2021). More specifically, scholars urge for multivariable models to include factors coming from multiple modalities of data sources such as clinical expertise or smartphone data (Chekroud et al. 2021; Gillett et al. 2020; Rost et al. 2023b; Sajjadian et al. 2023). Acknowledging the unique and insightful perspective of patients and informal caregivers (Kan et al. 2020), we argue that it is important that multivariable models include these experiences, including newly determined variables such as those found in the present study, to improve the mirroring of real‐world findings in research settings.

The current results also reflect findings of earlier research regarding the relationship between the therapeutic alliance, clinician, and client. Clients and informal caregivers find the therapeutic alliance important to treatment success and seem to attribute the alliance more to the clinician than to the client, as shown by this study and previous research (Bachelor 1995; McPherson et al. 2020). Additionally, a high‐quality therapeutic alliance is associated with better treatment outcomes (Arnow et al. 2013; Barber et al. 2014; Flückiger et al. 2012; Flückiger et al. 2018; Leibovich et al. 2020; Zilcha‐Mano et al. 2015). In our study, clients and informal caregivers sorted factors related to the therapeutic alliance more often with factors related to the clinician than to the client. This resulted in the location of the “Interaction client clinician” cluster being closer to “The clinician” cluster than to “The client” cluster on the map. The placement of the interaction‐cluster is especially meaningful, as the factors in this cluster do not semantically indicate responsibility or dominance of the clinicians. This means that the factors are not phrased in a way that indicates responsibility from the clinician. The participants must therefore consider the therapeutic alliance to be more related to the clinician than to the client. Previous research on expectations of clients on the therapeutic relationship also shows that clients believe a good therapeutic alliance is reliant on clinician‐facilitated activities, attitudes, and characteristics, emphasizing the dominance of the clinician (Bachelor 1995). For example, therapist qualities such as being non‐judgemental, listening carefully and including empathy are valued by clients in a positive therapeutic alliance (Bachelor 1995). In other qualitative studies, clients seem to use language for describing the (effect) of the therapeutic relationship that indicates dominance from the clinician (Rodenburg‐Vandenbussche et al. 2020; Shattell et al. 2007). Our results corroborate earlier findings that clients, and additionally informal caregivers, consider the therapeutic relationship more clinician‐driven (Bachelor 1995, 2013; McPherson et al. 2020; Rodenburg‐Vandenbussche et al. 2020; Shattell et al. 2007).

The present findings also indicate that clients and informal caregivers deem clinician′s adherence to good practice as a core of necessary factors for successful depression treatment. The factors in this cluster include organizational factors such as “short waiting lists” and “contact between the clinician and client′s other health care providers”, factors related to specific treatment elements such as “a relapse prevention plan” and “discussing practical tools on how to cope with depression”, and also factors related to adherence to clinical guidelines such as “discussing the diagnosis with the client” and “adequate psychoeducation for the client” (Akwa 2024; American Psychological Association 2019; Federatie Medische Specialisten 2013; National Institute for Health and Care Excellence 2022; NHG‐Richtlijnen 2019). The large diversity of statements can indicate that these factors were differently interpreted by participants. However, when inspecting the bridge value – a value between 0 and where lower values indicate that a factor was similarly grouped by participants and higher values indicate that a factor was less similarly grouped (Trochim and Kane 2005) – of the factors in this cluster, the bridge values are not higher than other statements on the map. Additionally, the expert group agreed that this cluster was most likely not placed in the middle of the map by chance, but because the factors in this cluster are experienced to be at the core or as prerequisites to successful treatment. This is also reflected in other literature, where a combination of practical aspects is considered important to successful treatment, next to client and therapist activities (Constantino et al. 2022; Finazzi and MacBeth 2022; Ladmanová et al. 2022; Løvgren et al. 2020; Marren et al. 2022; van Grieken et al. 2016). The current results add to these previous findings the notion that “adherence to clinical guidelines” is perhaps more broadly understood by clients and informal caregivers than by researchers. Additionally, the current results also add that these specific factors are likely seen by clients and informal caregivers to be at the core of successful treatment, and not only another important factor.

### 4.1 Recommendations

Building on these insights, the following main recommendations are proposed to enhance the integration of patient experiences and improve the effectiveness of treatment outcomes for depression.

Firstly, based on the current study, it could be valuable to include the identified factors in monitoring treatment progress by clinicians and clients to understand which factors they themselves consider to be pivotal for a successful treatment. Previous research has shown that the evaluation of treatment progress improves outcomes and reduces dropout (de Jong et al. 2021; Lambert et al. 2018). In our study, clients and informal caregivers also indicated they found this important (e.g., statement 56: “timely evaluation of the match between client and clinician” and statement 58: “monitoring (checking up on how the client is doing)”). Commonly used tools for monitoring are, e.g., the Outcome Questionnaire System (OQ System; Lambert et al. 2013) and the Partners for Change Outcome Measurement System (PCOMS; Miller et al. 2005). If clients and clinicians are interested in including more process‐related factors into account when discussing progress, the factors identified in this study could use the concept map and its factors to identify which factors are important to them and regularly check in on how those factors are being fulfilled. This might foster a climate of shared responsibility and improve monitoring rates and outcomes.

Secondly, clinicians who would like to discuss the (quality of the) alliance with clients should be aware of clients′ attribution of the alliance. Clients and informal caregivers find the therapeutic alliance important to treatment success and seem to attribute the alliance more to the clinician than to the client, as shown by this study and previous research, and additionally, high‐quality therapeutic alliance is associated with better treatment outcomes (Arnow et al. 2013; Bachelor 1995; Barber et al. 2014; Flückiger et al. 2012; Flückiger et al. 2018; Leibovich et al. 2020; McPherson et al. 2020; Zilcha‐Mano et al. 2015). However, clients and clinicians might have differing ideas about what a good therapeutic relationship entails (Bachelor 2013). Discussing the alliance and each′s attitudes can help clarify mutual expectations in the treatment room.

### 4.2 Strengths, Limitations, and Further Research

This study′s major strength is the use of the GCM method, bridging the gap between qualitative and quantitative research. The GCM method efficiently gathers insights inclusively in the brainstorming phase and allows for intraconceptual relations to be uncovered due to the sorting and rating phase. This dual approach underscores GCM′s capability to integrate and harmonize qualitative and quantitative data seamlessly.

Another strength of this study is the inclusion of clients and caregivers throughout the entire research process, including design and interpretation of results. It is important to include experienced experts in research as it can increase our understanding of the experience of depression and improve the connection between research and real‐life (Cuijpers 2019; Löffler‐Stastka et al. 2021). Clients and informal caregivers also indicate they are glad to be part of research (Tallon et al. 2011) and inclusive research is also stimulated by national patient associations (De Kennisagenda Depressie 2024). In our study, clients and informal caregivers were involved in setting up the study design, the collection and interpretation of data. Participants shared with us that they found their participation valuable beyond study results.

A limitation of this study is that there appear to be only a limited number of participants in the study. However, concept maps seem to become stable with the inclusion of around 25 participants (Rosas and Kane 2012) and concept mapping remains a method that is recommended to engage clients in improvement of clinical care (LaNoue et al. 2016). Even though a stable concept map was obtained, the diversity of participants was an obstacle in this study. No clients participating in the sorting and rating phase indicated that they experienced severe symptoms. Additionally, participants were relatively young and were generally highly educated. Further research could focus on including clients with higher symptom severity and further analyse different priorities between groups with varying severities. Additionally, a more varied sample size can be considered and compared to the current one in order to understand the impact of these limitations.

Another limitation of this study is that participants were sometimes difficult to characterize as clients or informal caregivers, as sometimes informal caregivers also experienced depressive symptoms and clients might have also provided care for others. This made any comparison between groups more complex. Additionally, we do not have information about how severe clients′ symptoms were in case treatment was in the past. Symptom severity during treatment might influence how important a factor is considered. Further research could focus on larger surveys like studies to understand the importance of the identified factors to specific groups.

## 5 Conclusion

Clients and informal caregivers were able to list key factors necessary for a successful depression treatment. These factors were clustered in ten concepts: “The client”, “Treatment process”, “Treatment organisation”, “Interaction client clinician”, “The clinician”, “Clinician′s adherence to good practice”, “Drug treatment”, “Pre‐condition”, “Supporting activities”, and “Supportive work and home life”. Our results support the notion that a multivariate and multimodal model can be useful in understanding depression treatment outcomes. In addition, we found that the therapeutic relationship is clinician‐driven in the clients′ and formal caregivers′ perspective, and we recommend that clinicians be aware of this attribution. Moreover, clients and informal caregivers consider a plethora of factors to be at the core of successful depression treatment that include factors related to treatment organization, specific treatment elements and adherence to clinical guidelines. Further research should focus on making continuing involvement a diverse group of experienced experts and instantiate and test the factors found in this study. The current findings could serve clients and clinicians as a guide to help identify what individual clients find important for treatment success and to help discuss these factors.

## Conflicts of Interest

The authors declare no conflicts of interest.

## Supporting information

Appendix A new.

Appendix B new.
