# Supplementary material for: Ingredients for Success: What Clients and Informal Caregivers Value in Depression Treatment A Concept Mapping Study
Source: J Clin Psychol. 2026 Jan 20;82(4):594–608. doi: 10.1002/jclp.70092 (PMC12965048; doi:10.1002/jclp.70092)

**Appendix D**

**Figure S2**

Go-zone comparing average statement importance having received treatment or not


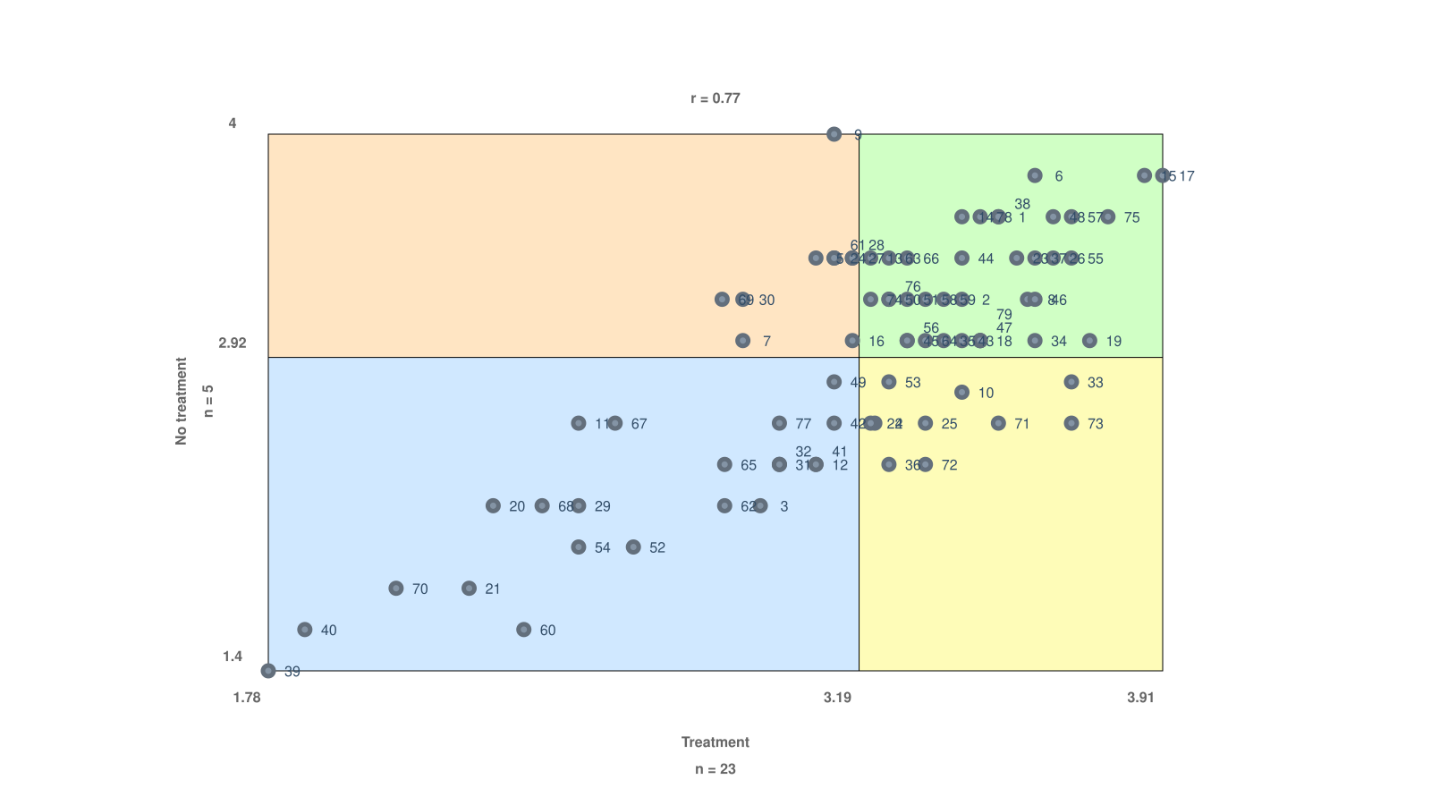

Supplement: Supplementary file 2 — Appendix B new. [file JCLP-82-594-s001.docx]
